# Assessment of Anti-Müllerian Hormone Level in Management of Adolescents with Polycystic Ovary Syndrome

**DOI:** 10.4274/jcrpe.2338

**Published:** 2016-03-01

**Authors:** Fatma Dursun, Ayla Güven, Metin Yıldız

**Affiliations:** 1 Ümraniye Education and Research Hospital, Clinic of Pediatric Endocrinology, İstanbul, Turkey; 2 Göztepe Education and Research Hospital, Clinic of Pediatric Endocrinology, İstanbul, Turkey; 3 Prof. Dr. Güven and Dr. Dursun have equal rights in this study.

**Keywords:** Polycystic ovary syndrome, adolescents, anti-Müllerian hormone, treatment

## Abstract

**Objective:**

This study was oriented to investigate the benefit of anti-Müllerian hormone (AMH) level in the management of polycystic ovary syndrome (PCOS). To assess the impact of metformin and oral contraceptives (OC) on serum AMH levels in a cohort of adolescents with PCOS.

**Methods:**

Forty-nine adolescents with PCOS were recruited to the study. Twenty-nine patients without insulin resistance were treated with OC (group 1), and 20 patients with insulin resistance were treated with metformin and OC (group 2). AMH and androgen levels were measured prior to and 6 months after the initiation of treatment.

**Results:**

AMH levels were significantly decreased with treatment in both group 1 (p=0.006) and group 2 (p=0.0048). There was a significant correlation between pre- and post-treatment AMH and left ovarian volume (pretreatment: rho=0.336, p=0.018; post-treatment: rho=0.310, p=0.034).

**Conclusion:**

This study investigated two different treatment regimens in adolescents with PCOS and revealed that AMH levels decreased with treatment. AMH levels were correlated with ovarian volume.

**WHAT IS ALREADY KNOWN ON THIS TOPIC?**

Anti-Müllerian hormone (AMH) levels decreased with treatment in adult patients with polycystic ovary syndrome (PCOS).

**WHAT THIS STUDY ADDS?**

This study revealed that AMH levels decreased with treatment in adolescent patients with PCOS.

## INTRODUCTION

Polycystic ovary syndrome (PCOS) is the most common endocrinopathy in women of reproductive age (1). PCOS is characterized by hyperandrogenism and oligomenorrhea, and is also highly associated with obesity and insulin resistance (IR) (1). Hyperandrogenism is the key element of the physiopathology responsible for the interruption of physiologic feedback mechanisms that are fundamental for the establishment of ovulatory cycles, which leads to chronic anovulation. However, primary ovarian dysfunction, and disorders of the production and action of growth factors and anti-Müllerian hormone (AMH) are other mechanisms associated with possible changes in follicular recruitment and development (2).

AMH is produced in the granulosa cells of early developing follicles. AMH expression in the ovary starts at the end of the third trimester of pregnancy, although its levels at birth can be almost undetectable. Serum levels of AMH increase after puberty, probably as a result of follicular growth, and it remains detectable until the end of ovarian activity. Serum levels of AMH were higher in patients with PCOS than in women with normal cycles in several studies (3,4). This hormone is thought to reflect the continuous, non-cycling growth of small follicles in the ovary. The level of AMH is not influenced by fluctuations in other reproductive hormones, and its level does not change throughout the menstrual cycle, which makes it a promising marker (5). Women with PCOS are usually treated with an oral contraceptive (OC), and women with PCOS who are obese and have IR might benefit from treatment with metformin. Treatment with OC is known to normalize menstrual function and to ameliorate hirsutism, although the effects on IR are controversial. There are scant data available on the impact of these treatments on serum AMH levels in women with PCOS. In the studies by Streuli et al (6) and Somunkıran et al (5), AMH levels did not decrease with OC use; conversely, Panidis et al (1) reported reduced AMH levels with OC (35 mg ethinylestradiol and 2 mg cyproterone acetate). Many studies have demonstrated that AMH levels decrease with metformin treatment (3,7). However, to our knowledge, no studies have evaluated AMH levels with metformin and OC treatment in adolescent patients with PCOS.

The objective of this study was to evaluate whether treatment with OC and OC plus metformin would reduce AMH levels in adolescents with PCOS.

## METHODS

### Patients

The study was conducted with 49 adolescent patients (aged 13-17.5 years) who were being followed-up for PCOS in Göztepe Education and Research Hospital, Pediatric Endocrinology Clinic patients who had been recently diagnosed as having PCOS were divided into two groups, those receiving OCs (group 1) and those receiving OCs plus metformin (group 2), and their treatments were evaluated. G power 3.1.4 was used for the sample size, which was calculated as 54 to give a power of 0.95 (1-β probability of error), effect size f: 0.5, and α probability of error: 0.05.

Thirty patients were recruited for each group at the start of the study but 9 were excluded due to treatment incompliance, and 2 due to failure to report for follow-up visits. Thus, group 1 included only 29 patients and group 2 comprised 20. This was planned as a two-stage prospective study; the study compared AMH, androgen levels, and pelvic ultrasonography (USG) before and after treatment, as well as between the two groups.

PCOS diagnosis was based on the Rotterdam (8) criteria (European Society of Human Reproduction and Embryology/American Society of Reproductive Medicine Consensus Workshop group). Accordingly, patients who met at least 2 of the 3 criteria below were included in the study (8). 1) Oligomenorrhea (cycle interval >45 days or amenorrhea (absent menses >3 months; 2) evidence of clinical and/or biochemical hyperandrogenism; and 3) polycystic ovaries (presence of more than 10 follicles with a diameter of at least 2-9 mm and ovarian volume more than 10 cm3 with USG). In addition, if at least 2 years had passed since the last menstruation, this was also considered as a diagnostic criterion for PCOS (8). Patients who were treated for hirsutism over the past 6 months were excluded. Further exclusion criteria included current chronic disease and drug use. For the differential diagnosis of hirsutism, patients with congenital adrenal hyperplasia, hyperprolactinemia, hypothyroidism, adrenal and ovarian tumors, or Cushing’s syndrome were also excluded. The standard adrenocorticotropic hormone test (Synacthen 0.25 mg/1 mL; Novartis Pharma, Rueil-Malmaison, France) was performed to exclude congenital adrenal hyperplasia in all patients with 17-hydroxy-progesterone (17-OHP) levels >2 ng/mL.

Approval from the hospital’s Scientific Research Evaluation Commission (approval no. 26.01.2011/9/A) and written consents from the patients and their relatives were received for the study.

### Method

Patients who presented to our clinic who had previously been diagnosed as having PCOS were measured pre-treatment and at least 6 months post-treatment (Pt) for AMH, total testosterone, 1,4 androstenedione (AS), dehydroepiandrostenedione sulfate (DHEAS), sex-hormone binding globulin (SHBG), insulin, glucose, prolactin (PRL), luteinizing hormone (LH), and follicle stimulating hormone (FSH) levels. Each patient was evaluated for age, menarche age, body weight, height, body mass index (BMI), hirsutism and menstruation disorders. From these measurements, BMI was calculated using the body weight (kg)/height (m2) formula. Based on the resulting BMI, patients with values above the 95th percentile of the age- and sex-matched curve were considered as obese in accordance with Bundak et al’s (9) data. Ferriman-Gallwey scores (FGS) greater than >8 were considered as clinical hyperandrogenism (10). All blood samples were collected between the second and fifth days of menstruation, in the morning after an overnight fast. For subjects with oligomenorrhea, blood was collected regardless of day, again in the morning after an overnight fast.

Prior to treatment initiation, each patient underwent oral glucose tolerance tests (OGTT) to investigate IR and to establish whether the patient had type 2 diabetes mellitus (DM). The test was performed by administering 1.75 g/kg (75 g) oral glucose following a 12-hour overnight fast.

OGTT-based diagnoses of IR, glucose tolerance, and type 2 DM were established based on the American Diabetes Association (ADA) criteria (11). Patients with normal OGTT results were started on OC only (ethinyl estradiol 0.03 mg+drospirenone 3 mg) (group 1), while those with impaired glucose tolerance and IR with OGTT were started on an OC and metformin combination (2000 mg/day, 2 doses) (group 2). Clinical and biochemical evaluations were repeated at month 3, and after 6 to 11 months of treatment. Trans-abdominal pelvic ultrasound was used to measure ovarian size and to assess the presence of PCO. All ultrasounds were performed by the same radiologist. Ovarian volume was calculated as V: width (mm) x length (mm) x thickness (mm) x 0.523/1000 (mL). Polycystic ovary was diagnosed if the ovarian size was larger than 10 cm3 or in the presence of more than 10 peripheral cysts with diameters of 2-9 mm (12,13).

LH (IU/L), FSH (IU/L), total testosterone (TT, ng/mL), DHEAS (DHEASO4, mcg/dL), insulin, and PRL were analyzed at the central laboratory of our hospital using an immunoenzymatic method (device: Beckman Coulter, DXI 800 USA). AS (ng/mL), 17-OHP (ng/mL) were studied using radioimmunoassay with the Immunotech (Beckman Coulter) kit and an ICN ISO DATA Gamma Counter, SHBG (nmol/L) was studied using a chemiluminescence assay with a Siemens kit and Siemens Immulite 2000XPI analyzer at the Gelişim Laboratory.

AMH (ng/mL) was studied by the Gelişim Laboratory using enzyme-linked immunosorbent assay (ELISA) with an Immunotech Gen II (A73818) Beckman Coulter kit and a Pasteur ELISA reader. The minimum sensitivity of the kit was 0.08 ng/mL. The intra-assay variability coefficient was 5.4% for 4.42 ng/mL and 3.6% for 14.03 ng/mL, and the inter-assay variability coefficient was 5.6% for 4.42 ng/mL and 4.5% for 14.03 ng/mL.

### Statistical Analysis

The results were transferred to ‘Statistical Package for the Social Sciences 15.0 for Windows’ software, which was used for the descriptive analyses of the study group. The Shapiro-Wilk test was used to establish whether the data were normally distributed. Insulin, Pt-insulin, Pt-LH, Pt-FSH, Pt-E2, Pt-SHBG, Pt-right ovary, and Pt-left ovary without normal distribution were expressed as median (IQR) and other data with normal distribution were expressed as mean ± standard deviation values.

The pre- and Pt data differences between groups 1 and 2 were studied using the independent samples t-test for data with normal distribution and Mann-Whitney U test for data without normal distribution.

The differences between pre- and Pt measurements of patients in groups 1 and 2 were calculated separately using the Paired Sample test for data with normal distribution and Wilcoxon’s signed-rank test for data without normal distribution. The difference in AMH values before and after treatment was designated as delta AMH. Correlations between the data were calculated using Spearman’s correlation (rho and p provided). The results were expressed with 95% confidence interval and statistical significance was set at p<0.05.

## RESULTS

The clinical characteristics of the study population are summarized in Table 1. With the exception of SHBG, which was significantly higher in group 1, groups 1 and 2 did not differ significantly in respects to pre-treatment LH, FSH, E2, AMH, AS, TT, and DHEAS. Following treatment, no significant differences were noted between the groups regarding hormonal values or AMH (Table 1).

**Figure 1:**
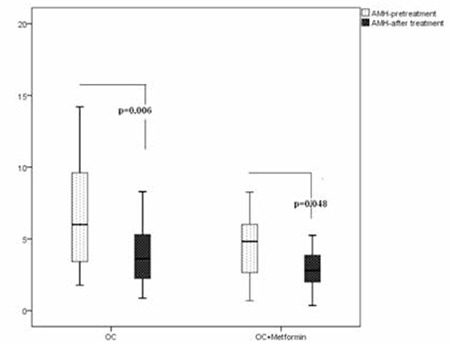
Box and whisker plots depicting the pre and post-treatment of serum anti-Müllerian hormone levels in patients of group 1 and group 2. Solid lines inside boxes depict the median anti-Müllerian hormone level, whereas the upper and lower limits of the boxes and whiskers indicate 75th, 25th, and 95th, and 5th percentiles. AMH: anti-Müllerian hormone, OC: oral contraceptive

Patients’ pre- and Pt AMH and other androgen levels were compared between the groups (Table 1), and significant decreases of AMH only were observed for both groups (Figure 1).

AMH serum levels were significantly decreased in group 1 from 6.7±3.7 ng/mL at baseline to 4.3±3.1 ng/mL after 6 months of treatment (p=0.006). In group 2, AMH serum levels ranged from 4.7±3.07 ng/mL at baseline to 3.1±2.05 ng/mL after 6 months of treatment (p=0.048).

Delta AMH was used to determine whether AMH reductions were significant between the groups; no significant difference was found (p=0.315, t=1.017). SHBG increased significantly in both groups. TT levels decreased in both groups but without statistical significance. AS decreased significantly in group 2 (p=0.02).

**Figure 2:**
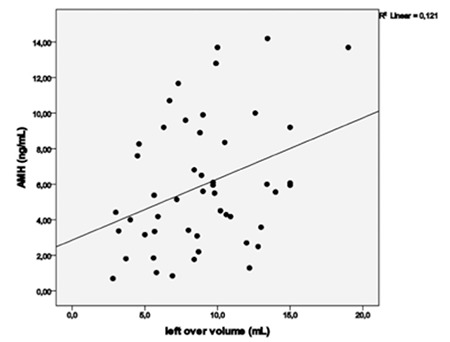
Relationships between serum anti-Müllerian hormone level and total left ovarian volume in patients with polycystic ovary syndrome. AMH: anti-Müllerian hormone

Evaluation of correlations between AMH, androgens, insulin, FGS and ovarian volumes did not yield any correlations between AMH, FGS, and androgens. There was only a significant correlation between AMH and left ovarian volume (rho=0.336, p=0.018) (Figure 2). DHEAS was correlated with AS (rho=0.347, p=0.026) and TT (rho=0.775, p<0.001).

Evaluation of correlations between Pt FGS, AMH, androgens, insulin, and ovarian volume demonstrated a correlation between AMH and left ovarian volume (rho=0.310, p=0.034). There was no correlation between Pt AMH, FGS, and androgens.

## DISCUSSION

This study aimed to investigate whether AMH was a good indicator for treatment and monitoring of adolescent patients with PCOS. For this purpose, levels of AMH and other androgens were studied in patients who were newly diagnosed as having PCOS, after which the patients were divided into two groups to investigate changes in AMH levels in groups and treated with OC or OC plus metformin.

Several studies have demonstrated increased levels of AMH in adult women and adolescent girls with PCOS (3,4,14,15,16). However, few studies have shown decreased levels of AMH, like other androgens, with metformin treatment and OC in adult women with PCOS (3). Moreover, no studies have reported a relationship between OC or OC plus metformin treatment and AMH levels in adolescents with PCOS. Therefore, our study is the first in the literature.

Studies on the relationship of AMH with insulin in PCOS are contradictory. Some studies found no correlation between insulin and AMH levels (17), whereas others identified a positive relationship between them (18). Tomova et al (19) found no relationship between AMH and insulin although a positive correlation between insulin and androgens was found. Pigny et al (4) identified no relationships. This is supported by the fact that there were no relationships between BMI and AMH in the present study. There was a negative relationship between AMH and insulin in our study but no relationship between BMI and AMH was found. Insulin may lead to hyperandrogenism by reducing SHBG production in the liver and also by increasing the amount of circulating free testosterone, a biologically active androgen (20). Likewise, our patients in the IR group had significantly lower SHBG compared with group 1.

The relationship between AMH and ovarian volume has been investigated in several studies because attempts are being made to ascribe AMH elevations in patients with PCOS to increased numbers of small follicles. A positive relation between AMH and mean ovarian volume was found in the study of Li et al (21). Healthy adolescents without PCOS but with polycystic ovary morphology also had higher AMH levels (22), which indicated that AMH was related to an increased follicle count. Hart et al (23) failed to demonstrate in a general adolescent study population that serum AMH was a reliable predictor of PCO morphology or for the presence of PCOS. However, we found a significant relationship between pre- and Pt AMH levels and left ovarian volume. Additionally, reductions in right and left ovarian volumes with treatment were noted.

Piltonen et al (3) were the first to demonstrate reduced levels of AMH with metformin treatment in adult women with PCOS. In the study by Tomova et al (19), 17 patients with PCOS were treated with metformin at 2550 mg/day and at the end of 6 months, correction of irregular menstruation and reduced AMH levels were shown in 13 patients; the remaining 4 had no clinical improvement and their AMH levels increased. Nascimento et al (2) observed significant reductions in insulin and testosterone with no changes in AMH levels. In the study by Somunkiran et al (5), AMH levels of 30 adult patients with PCOS were measured before and after 6 months of treatment with OC (35 mcg ethinyl estradiol+2 mg cyproterone acetate), with no significant changes detected; however, reduction in ovarian volume and follicle count were observed. Panidis et al (1) randomized adult subjects with PCOS into 3 groups and administered an OC regimen to group 1 (35 mcg ethinylestradiol+2 mg cyproterone acetate), another OC regimen to group 2 (30 mcg ethinylestradiol+3 mg drospirenone), and metformin to group 3 (2x850 mg). AMH levels decreased significantly only in group 1 with no significant changes in the other two groups. These are studies with adult subjects with PCOS but no studies in the literature have demonstrated AMH changes in adolescent subjects with PCOS. In the present study, we investigated changes in AMH levels with OCS and whether changes in AMH levels would differ in adolescent PCOS patients with IR when given OC plus metformin versus OC alone. AMH levels were significantly reduced in both groups but without a significant difference between the groups; metformin did not induce an additional alternation in AMH levels.

We did not measure free testosterone because free testosterone measurements are not very reliable, thus we preferred to measure total testosterone. TT levels decreased in both groups but without statistical significance. In our study, we found no correlation between AMH level and FGS or testosterone levels. In group 1, where FGS was not statistically significant, there was a decline on AMH and ovarian volumes. This finding indicates that the decline in AMH was independent of hyperandrogenism and was mostly related with ovarian volumes. Rosenfield et al (24) showed that AMH levels were independently related to ovarian androgenic function. In the absence of hyperandrogenism, moderate AMH elevation in women with normal-variant polycystic ovaries seems to indicate an enlarged oocyte pool (24).

One of the weaknesses of our study was that it did not incorporate a patient group treated with metformin alone because it was not possible to distinguish which agent was more effective in reducing AMH levels in group 2, although it is likely that AMH levels were decreased with OC only. A second weakness is the lack of a control group. This was because the approval of the local ethics board could not be obtained for healthy controls. Another limitation of our study was the inadequate number of patients.

In conclusion, the present study demonstrated that treatment reduced AMH levels in adolescents with PCOS and it was not associated with hyperandrogenism. AMH correlated with ovarian volume and both AMH and ovarian volume decreased after treatment. We think that AMH could be used instead of transabdominal pelvic USG, independent of hyperandrogenism. AMH seems to be a good parameter for monitoring adolescent patients with PCOS because it is easy to measure at any period during the cycle. However, in the absence of adequate relevant studies, larger studies are needed.

## Ethics

Ethics Committee Approval: Medeniyet University Göztepe Training and Research Hospital (Approval number: 26.01.2011/9/A),

Informed Consent: It was taken.

Peer-review: External peer-reviewed.

## Figures and Tables

**Table 1:**
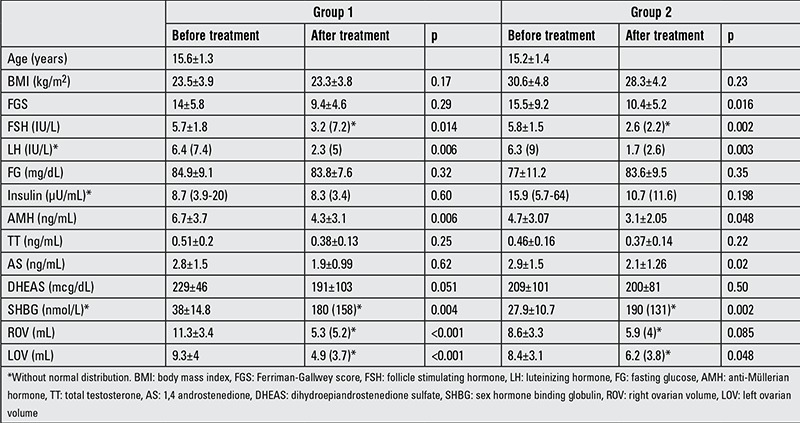
Clinical and hormonal characteristics of the patients with polycystic ovary syndrome at baseline and after-treatment
